# Investigating the association between anxiety symptoms and mood disorder in high-risk offspring of bipolar parents: a comparison of Joint and Cox models

**DOI:** 10.1186/s40345-019-0157-9

**Published:** 2019-10-18

**Authors:** Ruoxi Dong, George Stefan, Julie Horrocks, Sarah M. Goodday, Anne Duffy

**Affiliations:** 1 Department of Mathematics and Statistics, University of Guelph, 50 Stone Road East, Guelph, ON N1G 2W1 Canada; 2 Department of Psychiatry, University of Oxford, Warneford Ln, Oxford, OX3 7JX UK; 3 Department of Psychiatry, Queen’s University, 99 University Ave, Kingston, ON K7L 3N6 Canada; 4 Visiting Fellow, All Souls College, University of Oxford, High Street, Oxford, OX1 4AL UK

**Keywords:** High-risk, Offspring, Longitudinal, Bipolar disorder, Depressive disorder, Anxiety, Joint model, Survival analysis, Cox model, Measurement error

## Abstract

**Background:**

Anxiety is associated with mood disorders including bipolar disorder. Two statistical modelling frameworks were compared to investigate the longitudinal relationship between repeatedly measured anxiety symptoms and the onset of depression and bipolar disorder in youth at confirmed familial risk.

**Methods:**

Prospectively collected data on 156 offspring of a parent with confirmed bipolar disorder participating in the Canadian Flourish high-risk offspring longitudinal cohort study were used for this analysis. As part of the research protocol at approximately yearly visits, a research psychiatrist completed the HAM-A and a semi-structured diagnostic research interview following KSADS-PL format. Diagnoses using DSM-IV criteria were made on blind consensus review of all available clinical information. We investigated two statistical approaches, Cox model and Joint model, to evaluate the relationship between repeated HAM-A scores and the onset of major depressive or bipolar disorder. The Joint model estimates the trajectory of the longitudinal variable using a longitudinal sub-model and incorporates this estimated trajectory into a Cox sub-model.

**Results:**

There was evidence of an increased hazard of major mood disorder for high-risk individuals with higher HAM-A scores under both modelling frameworks. After adjusting for other covariates, a one-unit increase in log-transformed HAM-A score was associated with a hazard ratio of 1.74 (95% CI (1.12, 2.72)) in the Cox model compared to 2.91(95% CI (1.29, 6.52)) in the Joint model. In an exploratory analysis there was no evidence that family clustering substantially affected the conclusions.

**Conclusions:**

Estimated effects from the conventional Cox model, which is often the model of choice, were dramatically lower in this dataset, compared to the Joint model. While the Cox model is often considered the approach of choice for analysis, research has shown that the Joint model may be more efficient and less biased. Our analysis based on a Joint model suggests that the magnitude of association between anxiety and mood disorder in individuals at familial risk of developing bipolar disorder may be stronger than previously reported.

## Background

Family and adoption studies provide evidence that bipolar disorder is highly heritable with an estimated 60–85% of the risk related to genetic influences (Smoller and Finn 2003). Offspring of parents with bipolar disorder are therefore an informative high-risk group. High-risk offspring are at increased risk of developing both depressive and bipolar disorders and depressive disorders—particularly recurrent—form part of the bipolar phenotypic spectrum in family studies (Duffy et al. 2014, 2018; Mesman et al. 2013). For over two decades, we have been prospectively studying the offspring of well-characterized bipolar parents to describe the developmental trajectory of mood disorder development (Duffy et al. 2014, 2018). We and others (Hafeman et al. 2016) have shown that anxiety disorders and clinically significant anxiety symptoms are associated with and predict the onset of major mood disorder (Duffy et al. 2013, 2018; Nurnberger et al. 2011). In this manuscript, we compared two different statistical approaches to study the longitudinal relationship between repeatedly measured antecedent anxiety symptoms and the diagnosis of major depression or bipolar disorder in high-risk individuals.

A conventional and commonly adopted way to study this relationship would be to fit a Cox model with time to diagnosis as the outcome and with the repeatedly measured anxiety symptom scores as a time-varying predictor variable (Collett 2014). The Cox model assumes that predictor variables (also called covariates) are non-random, i.e. not subject to variability or uncertainty, as this can lead to bias in parameter estimates (Prentice 1982). However, it is very likely that anxiety symptom scores cannot be measured precisely and/or involve substantial variability around a conceptual true value, due to minute-by-minute fluctuations in anxiety, subjective assignment of clinical values, inter-rater variability, among other possibilities. This variability is often called “measurement error” when it occurs in a predictor variable, rather than in an outcome variable (Gustafson 2004). This is in contrast to covariates like sex or age whose values are known exactly. In addition, by default the Cox model assumes that time-varying covariates are constant between measurement times, a convention known as “Last Value Carried Forward”, and it is not well known whether clinical symptoms such as anxiety are in fact stable over short time periods.

Joint modelling was designed to utilize all available information in datasets that contain both longitudinal and survival components and to quantify the association between them (Schluchter 1992; Self and Pawitan 1992). Joint models (Henderson et al. 2000; Tsiatis and Davidian 2001; Rizopoulos 2012) accommodate measurement errors in repeatedly measured variables (Rizopoulos 2012) and do not assume the variables remain constant between measurements. A Joint model consists of two sub-models, a mixed-effects sub-model for the time-varying longitudinal data (e.g. anxiety symptom score), and a Cox sub-model for the time-to-event data (e.g. mood disorder). Conceptually, the Joint model first estimates the trajectory of the time-varying longitudinal variable, assuming that it follows a mixed-effects model. It then fits a Cox model using the estimated trajectory as a time-varying covariate (Rizopoulos 2012). In general, Joint models are more efficient compared to a conventional Cox model in which the longitudinal process is specified as a time-varying covariate (Gould et al. 2014).

In this paper, we compare the two approaches in estimating the association between anxiety symptom scores and the hazard of mood disorder diagnosis. This is an increasingly relevant methodological question, given the recognized need and increased interest in longitudinal study designs. First, anxiety scores were treated as time-varying covariates in a Cox model. Next the anxiety scores were modelled simultaneously with time to diagnosis in a Joint model. The effect of clustering within families was investigated using a frailty model.

## Methods

### Data background

The data used for this analysis were collected as part of the Flourish Canadian high-risk offspring longitudinal cohort study described in detail elsewhere (see Duffy et al. 2014, 2018). This study obtained ethics approval from the local Ottawa Independent Research Ethics Board and the Queen’s University Health Sciences Research Ethics Board (HSREB). Briefly, offspring ages 8 to 25 years were identified at baseline from parents with a confirmed Bipolar I or II diagnosis. Parents were assessed by a research psychiatrist using SADS-L format interviews. Diagnosis was based on blind consensus review of all available research and clinical evidence using best estimate procedure, by two additional research psychiatrists. Eligible offspring were those who were ages 5 to 25 years without major neurological or medical illness. All eligible assenting/consenting offspring in each family were admitted to the study (i.e. no limit per family). At baseline parents were interviewed about the developmental and clinical history of each child and families were invited to provide copies of any prior clinical or psychoeducational reports. All offspring from identified families completed repeated semi-structured research interviews, following KSADS-PL format, conducted by a research adolescent psychiatrist. These offspring have been followed up periodically since 1995. Research visits were conducted when the offspring were well or in remission and at their best level of functioning and not during acute episodes of illness. This is an ongoing dynamic cohort study, and therefore eligible offspring are enrolled at different ages and times and followed prospectively from that point forward. Therefore, each offspring has a variable age at entry and duration of follow-up.

The original longitudinal cohort of high-risk offspring comprised 298 individuals in 121 families. There were multiple offspring per family in some cases. Offspring who had no recorded Hamilton Anxiety (HAM-A) scores (Hamilton 1959) or only had HAM-A scores recorded after their diagnosis, were excluded from this analysis, as they contributed no information on the predictability of the outcome, leaving 156 offspring clustered in 85 families. Characteristics of offspring in the full dataset and subset with HAM-A scores are shown in Table 1.

**Table 1:** Characteristics of offspring in the full dataset and the subset with HAM-A scores

Characteristics	Full data set (%)	Subset with HAM-A scores (%)
Total number: n	298	156
Outcome (either major mood or bipolar disorder): no	195 (65.4)	125 (80.1)
Outcome (either major mood or bipolar disorder): yes	103 (34.6)	31 (19.9)
Bipolar disorder: no	258 (86.6)	150 (96.2)
Bipolar disorder: yes	40 (13.4)	6 (3.8)
Gender of offspring: female	178 (59.7)	85 (54.5)
Gender of offspring: male	120 (40.3)	71 (45.5)
Parent Lithium response: positive	132 (44.3)	62 (39.7)
Parent Lithium response: negative	166 (55.7)	94 (60.3)
SES 1	1 (0.3)	1 (0.6)
SES 2	7 (2.3)	7 (4.5)
SES 3	30 (10.1)	19 (12.2)
SES 4	105 (35.2)	51 (32.7)
SES 5	154 (51.7)	78 (50)
Parent onset age, years: median	24.19	25.01
Median age at entry	16.38	15.00
Median age at event or censoring	24.62	23.63
Median number of visits	3	2

Repeated anxiety symptoms were measured using the Hamilton Anxiety Rating Scale (Hamilton 1959). This scale includes 14 items that address both psychiatric and somatic anxiety symptoms. The total anxiety score ranges from 0 to 56. A total score of 0 to 7 is considered the normal range for anxiety level in healthy individuals, 8 to 14 indicates mild anxiety, 15 to 23 indicates moderate anxiety, and 24 to 56 indicates severe anxiety (Matza et al. 2010). Subjects were assessed by a research psychiatrist approximately annually for up to 20 years. The subject’s age at the time of each assessment was also recorded.

The event of interest for this analysis was the first occurrence of meeting full diagnostic criteria for either a major depressive or a bipolar disorder diagnosis (i.e. bipolar I, bipolar disorder II, or bipolar disorder not otherwise specified). The time scale for the analysis was age in years, so that a time of 0 represents birth. In the data set used for analysis, 31 individuals experienced the event of interest before the end of the study period. The remaining 125 individuals who did not develop the outcome by last assessment are referred to as “censored” individuals.

The total HAM-A score, which varies over time, was the primary predictor variable of interest. Other variables selected for this analysis, thought to be potential confounders, include sex of offspring, parent long-term lithium response (determined by research protocol), parent socio-economic status (SES) score, parent onset age, and subject’s age at initial interview. SES was calculated based on the participant’s parents’ education levels and occupation at the time of recruitment using the Hollingshead SES Scale (Hollingshead 1975). This ordinal score ranged from 1 to 5, with 1 representing the lowest and 5 indicating the highest SES level. The time scale used for this analysis was age in years, with time 0 representing an individual’s time of birth.

Table 1 shows that the subset with recorded HAM-A scores prior to the outcome (onset of a major depressive or bipolar disorder) or censoring, experienced fewer events (major mood or bipolar disorder) than the whole data set (19.9% versus 34.6%). The proportion of offspring diagnosed with bipolar disorder is also greater in the full dataset (13.4%) than the subset used for analysis (3.8%).

**Fig. 1:**
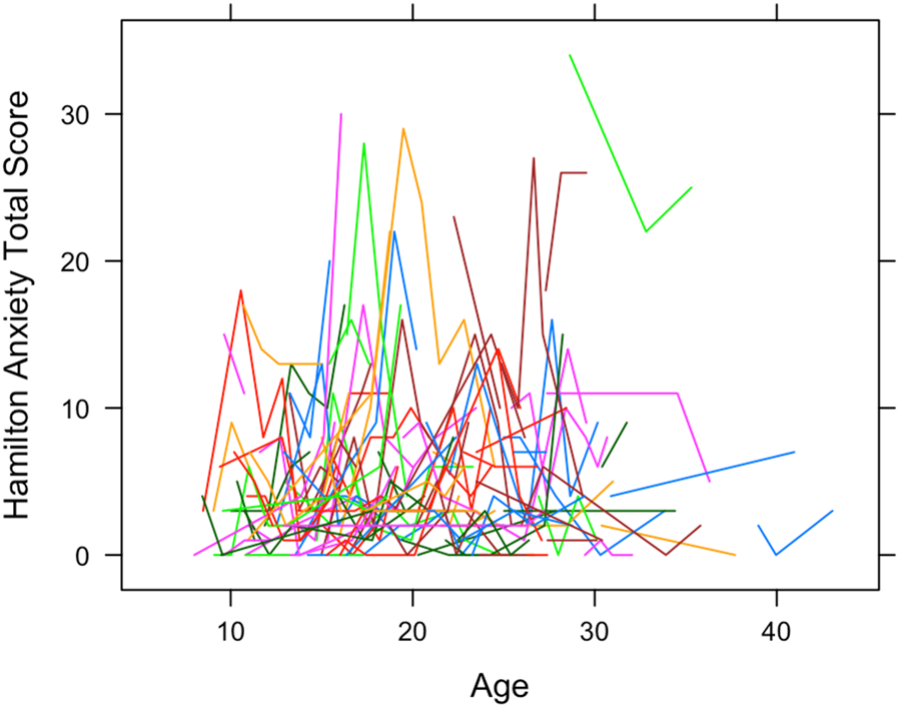
Hamilton anxiety scores plotted against age of assessment. Each line represents a unique individual (n = 156)

Figure 1 shows HAM-A scores plotted against age of assessment. Each line represents a unique individual. HAM-A scores measured after diagnosis were excluded, as they have no predictive value. Individuals exhibited substantial variability in HAM-A scores over time as evidenced by the lack of smoothness in the lines. Most subjects had a recorded HAM-A total score below 14, indicating mild symptoms.

### Cox models

A conventional method to model data with both longitudinal and time-to-event components is to fit a Cox model with the longitudinal component specified as a time-varying covariate. Suppose that there are $n$ subjects under observation, and that both longitudinal data and time-to-event data are available for these subjects.

A Cox model with several time-fixed covariates and a single time-varying covariate can be represented as

1 $$h_{i} \left( t \right) = h_{0} \left( t \right){ \exp }\left\{ {w_{i1} \gamma_{1} + w_{i2} \gamma_{2} + \ldots + w_{iq} \gamma_{q} + \alpha y_{i} \left( t \right)} \right\}$$

where $h_{i} \left( t \right)$ is the hazard of the event (here major mood diagnosis) for individual $i$ at time $t$ and $h_{0} \left( t \right)$ represents a baseline hazard function that is left unspecified (Collett 2014). Time-fixed covariates are denoted $w_{i1} , w_{i2} , \ldots , w_{iq}$ with associated regression parameters $\gamma_{1} , \gamma_{2} , \ldots ,\gamma_{q}$. The time-varying covariate is represented by $y_{i} \left( t \right)$ and the associated scalar parameter $\alpha$ indicates the level of association between the observed longitudinal measurements (anxiety symptoms) and the hazard of the event (diagnosis of major mood disorder). At any given time point $t$, the hazard ratio for an event occurrence is ${ \exp }\left( \alpha \right)$ for a unit increase in $y_{i} \left( t \right)$.

Generally, measurements on $y_{i}$ are only available at observed measurement times $t_{ij}$ which likely do not correspond to event times. Yet to estimate the Cox model, we need measurements on the time-varying covariate at all the event times, even the event times of other people. This means that the survival model must make assumptions about the value of the time-varying covariate in between the observed measurement times. A popular technique used to fill in the missing values between observation times is called Last Value Carried Forward (LVCF) method. As its name suggests, it uses the last available observed value of $y_{i}$ before the required time $t$. This method will be used when fitting the Cox model with time-varying covariate below. The Cox models in this analysis were fitted using the *coxph* function from the *R* package *survival* (R Core Team 2013).

### Joint models

The purpose of a Joint model is to assess the association between repeatedly measured longitudinal predictors and a time-to-event outcome. The Joint model framework is comprised of two linked sub-models: the longitudinal sub-model and the Cox sub-model.

#### Longitudinal sub-model

The longitudinal data for individual $i$, $y_{i} \left( t \right)$, is modelled as an unobserved trajectory over time $t$, $m_{i} \left( t \right)$, plus random errors, $\varepsilon_{i} \left( t \right)$. The trajectory $m_{i} \left( t \right)$ is allowed to depend on predictor variables, $x_{i1} ,x_{i2} ,\, \ldots , x_{ip} ,$ a random intercept, $b_{0i}$ and random slope $b_{1i}$. All random quantities are assumed to be independent and normally distributed. The model can be represented as:

2 $$\left\{ {\begin{array}{*{20}l} {y_{i} \left( t \right) = m_{i} \left( t \right) + \varepsilon_{i} \left( t \right),} \\ {m_{i} \left( t \right) = x_{i1} \left( t \right)\beta_{1} + x_{i2} \left( t \right)\beta_{2} + \ldots + x_{ip} \left( t \right)\beta_{p} + b_{0i} + tb_{1i} ,} \\ {b_{i} \sim N\left( {0, D} \right),} \\ {\varepsilon_{i} \left( t \right)\sim N\left( {0, \sigma^{2} } \right).} \\ \end{array} } \right.$$

Quantities which are unknown and must be estimated include the regression parameters $\beta_{1} , \beta_{1} , \ldots ,\beta_{p}$, covariance matrix $D$, and variance $\sigma^{2}$.

#### Cox sub-model

The Cox sub-model has the form:

3 $$\begin{aligned} h_{i} \left( t \right) &= h_{0} \left( t \right)\exp \left\{ \vphantom{{+ w_{iq} \gamma_{q} + \alpha m_{i} \left( t \right)}}{w_{i1} \gamma_{1} + w_{i2} \gamma_{2} + \ldots }\right.\\ & \quad \left.{+ w_{iq} \gamma_{q} + \alpha m_{i} \left( t \right)} \right\}, t > 0 \end{aligned}$$

with notation defined above. The hazard ratio for a one unit increase in $w_{ij}$ is given by ${ \exp }\left( {\gamma_{j} } \right)$. By including $m_{i} \left( t \right)$, the unobserved trajectory of the longitudinal data, in the Cox sub-model, we have linked the longitudinal observations with the survival model. The parameter $\alpha$ represents the association between the hazard of the outcome and the trajectory, and is of primary interest in our analysis. The hazard ratio for a unit increase in $m_{i} \left( t \right)$ at time $t$ is given by ${ \exp }\left( \alpha \right)$.

The baseline hazard function can be left unspecified or modelled. However, Hsieh et al. (2006) have suggested that within the Joint modelling framework, leaving the baseline hazard function unspecified may lead to an underestimation of the standard errors of the covariate estimates. To avoid this, we specified that the hazard was constant within five equally-spaced time intervals. All Joint models were fitted using the *R* package *JM* (Rizopoulos 2010).

## Results

### Cox model

The conventional and commonly adopted approach to study the relationship between repeatedly-measured anxiety scores and time to diagnosis of major depressive or bipolar disorder would be to fit a Cox model with time to diagnosis as the outcome and with the repeatedly measured HAM-A scores as a time-varying covariate. We first present this model, which also includes offspring sex, parent lithium response, SES, parent age of onset and subject’s age at initial interview. The proportions of individuals in SES categories 1, 2, and 3 were small, which makes estimation difficult. Therefore, for the purpose of this analysis, SES 1, 2, and 3 were combined and represented as SES_123 in the model. The most common status of SES (SES 5) was used as the reference category. In *R*, the Cox model by default assumes that the HAM-A scores are constant from one time of assessment to the next (i.e. uses a LVCF approach). We log-transformed HAM-A scores in order to achieve normality, which is necessary for the joint model. Hereafter the transformed HAM-A scores are referred to as *log*HAMA.

Results obtained from fitting a Cox model are shown in Table 2. We observed that a single unit increase in the time-dependent covariate *log*HAMA increased the hazard of diagnosis of major depressive or bipolar disorder by 74% (estimate = 0.555, HR = 1.742, HR 95% CI (1.118, 2.714), p-value = 0.014), after adjusting for other variables in the model. The hazard of major mood disorder diagnosis was not significantly affected by subject sex (p-value = 0.188), parent lithium response (p-value = 0.379), parent SES (p-value = 0.717), parent age of onset (p-value = 0.985), or subject’s age at initial interview (p-value = 0.519), after adjusting for other variables in the model.

**Table 2:** Cox model (n = 156) with 95% confidence interval

Variable	Estimate (95% CI)	p-value
*log*HAMA	0.555 (0.111, 0.999)	0.014
Female offspring	0.527 (− 0.258, 1.312)	0.188
Male offspring	*	-
Lithium responder parent	0.360 (− 0.443, 1.162)	0.379
Lithium non-responder parent	*	-
SES 123	− 0.060 (− 1.208, 1.087)	0.717^a^
SES 4	0.319 (− 0.506, 1.143)	
SES 5	*	
Parent onset age	0 (− 0.040, 0.040)	0.985
Age at initial interview	0.031 (− 0.063, 0.125)	0.519

* = Reference level

^a^p-value obtained by a partial likelihood ratio test on 2 degrees of freedom; other p-values obtained by Wald tests

Tests of the proportionality assumption for each time-fixed covariate were carried out using the *cox.zph* (Therneau 2015) function in *R*. No evidence against the proportionality assumption in the Cox model was found.

The previous model assumes that all observations are independent. Our dataset included 156 individuals in 85 families and individuals from the same family are likely dependent. The effect of family clustering was investigated using a Cox model with frailty term, which accounts for clustering (Table 3). The results are quite similar to the Cox analysis without frailty (Table 2). This suggests that there is a strong relationship between HAM-A scores and diagnosis of mood disorder, even after taking account of familial clustering. Note that these analyses using Cox models do not properly account for measurement error.

**Table 3:** Cox model with frailty (n = 156). 95% CI = 95% confidence interval

Variable	Estimate (95% CI)	p-value
$log{\text{HAMA }}$	0.642 (0.165, 1.118)	0.008
Female offspring	0.539 (− 0.279, 1.357)	0.200^a^
Male offspring	*	
Lithium responder parent Lithium non-responder parent	0.463 (− 0.471, 1.397) *	0.330
SES 123	− 0.177 (− 1.479, 1.125)	0.135^a^
SES 4	0.410 (− 0.573, 1.392)	
SES 5	*	
Parent onset age	− 0.006 (− 0.052, 0.041)	0.810
Age at initial interview	0.019 (− 0.087, 0.125)	0.720
Family frailty	-	0.250

* = Reference level

^a^p-value obtained by a partial likelihood ratio test on 2 degrees of freedom; other p-values obtained by Wald tests

### Joint model

A longitudinal sub-model with random intercept and slope was fitted to the *log*HAMA scores, with time-varying predictor variable offspring age of HAM-A assessment; and time-fixed predictor variables sex, parent lithium response, SES, parent age of onset, and age at initial interview. All possible two-way interactions between variables were also examined and none were found to be significant at the 5% level. Therefore, they were omitted from this model. A Cox sub-model was fit with time-fixed covariates offspring sex, parent lithium response, SES, parent onset age, and age at initial interview. The estimated trajectory of *log*HAMA was also included as a time-varying predictor variable.

The fitted Joint model is summarized in Table 4. The top half of the Table shows the results from the longitudinal sub-model, which we now describe. The *log*HAMA scores were found to increase by 0.039 units per year (estimate = 0.039, 95% CI (0.013, 0.065), p-value = 0.003). Female offspring are more likely to experience higher $log{\text{HAMA}}$ scores than male offspring (estimate = 0.249, 95% CI = (0.008, 0.489), p-value = 0.043). Those with a lithium responder parent were found to have lower *log*HAMA scores by 0.262 units (estimate = − 0.262, 95% CI = (− 0.516, − 0.007), p-value = 0.044) compared to those whose parent did not respond to prophylactic or long-term lithium treatment. No difference in *log*HAMA scores were found between SES levels (p-value = 0.106) and parent onset age (p-value = 0.914). Lastly, age at initial interview had no significant effect on scores *log*HAMA (p-value = 0.128).

**Table 4:** Joint model (n = 156) with 95% confidence interval

Sub-model	Variable	Estimate (95% CI)	p-value
Longitudinal	Age of HAM-A Measurement	0.039 (0.013, 0.065)	*0.003*
	Female offspring	0.249 (0.008, 0.489)	*0.043*
	Male offspring	*	–
	Lithium responder parent	− 0.262 (− 0.516, − 0.007)	*0.044*
	Lithium non-responder parent	*	–
	SES 123	0.372 (0.013, 0.731)	0.106^a^
	SES 4	0.199 (− 0.070, 0.467)	
	SES 5	*	
	Parent onset age	− 0.001 (− 0.014, 0.012)	0.914
	Age at initial interview	0.023 (− 0.053, 0.007)	0.128
Cox	Female offspring	0.500 (− 0.269, 1.269)	0.203
	Male offspring	*	–
	Lithium responder parent	0.431 (− 0.398, 1.260)	0.308
	Lithium non-responder parent	*	–
	SES 123	− 0.299 (− 1.444, 0.845)	0.274^a^
	SES 4	0.392 (− 0.454, 1.238)	
	SES 5	*	
	Parent onset age	− 0.006 (− 0.050, 0.038)	0.797
	Age at initial interview	− 0.115 (− 0.191, − 0.039)	*0.003*
	Estimated trajectory of *log*HAMA	1.067 (0.258, 1.875)	*0.010*

Italic values indicate significance of p-value (p < 0.05)

* = Reference level

^a^p-value obtained by a partial likelihood ratio test on 2 degrees of freedom; other p-values obtained by Wald tests

The results from the Cox sub-model are shown in the lower half of Table 4. The association parameter, $\alpha$, measures the effect of the estimated trajectory of *log*HAMA on the risk of diagnosis. The only significant effects in the Cox sub-model were the age at first interview (estimate = − 0.115, HR = 0.891, HR 95% CI (0.826, 0.962), p-value = 0.003), and the association with the estimated trajectory of the *log*HAMA score (estimate = 1.067, HR = 2.907, 95% CI (1.294, 6.521), p-value = 0.010).

## Discussion

Both the Cox model and the Joint model found evidence of a significant association between clinically assessed anxiety symptoms (HAM-A scores) in this sample of well or remitted high-risk offspring of bipolar parents and the development of a major mood disorder. Further, there was evidence that anxiety symptoms increased with increasing age, were higher among females and were lower among offspring of parents with a lithium responsive subtype of bipolar disorder. These findings are consistent with our prior findings and the extant literature showing a predictive association between clinically significant anxiety symptoms or anxiety disorders and subsequent mood disorder in high-risk offspring of bipolar parents (Duffy et al. 2013, 2014, 2018; Nurnberger et al. 2011). Further, we have shown that the lithium responsive subtype of bipolar disorder tends to have full or very good quality of remission between mood episodes and less comorbidity with anxiety disorders—phenotypic characteristics that appears to breed true in affected family members (Duffy et al. 2018; Grof et al. 1983, 1994, 2009).

In this analysis we focused on comparing two different statistical approaches to study the association between repeatedly measured HAM-A scores and the diagnosis of major mood disorder; namely, a Cox model with time-varying covariate was compared to the Joint modelling approach. There was an increased hazard of diagnosis for subjects with higher *log*HAMA scores under both modelling frameworks. In the Cox model, the effect of *log*HAMA was significant (estimate = 0.555, HR = 1.742, HR 95% CI (1.118, 2.71), p-value = 0.014), after controlling for other variables in the model. In the Joint model, *log*HAMA was found to have a much larger effect on diagnosis (estimate = 1.067, HR = 2.907, HR 95% CI (1.294, 6.521), p-value = 0.010), after controlling for other variables in the model.

Under the Cox model, *log*HAMA scores were assumed to be measured without error and constant between two consecutive assessments. The Joint model properly accounts for error in the measurement of *log*HAMA scores and models *log*HAMA scores as a smooth trajectory. The smaller effect in the Cox model is consistent with the measurement error literature (Rizopoulos 2012; Gustafson 2004). The observed large difference in magnitude of effect underscores the utility of using the Joint model rather than the Cox model for repeated predictor and time-to-event data.

The estimation of association between longitudinal and survival processes using the Cox model with time-varying covariate can result in bias, as the model ignores any measurement errors in the repeated measures (Asar et al. 2015) and assumes that the covariate values are constant between measurements. Advantages of the Joint modelling approach are the correct treatment of measurement error and the appropriate handling of the intermittently observed time-dependent covariate information, which can reduce bias in the estimation of the relationship between longitudinal and time-to-event processes (Asar et al. 2015). The complexity of calculations are much higher with the Joint model than Cox regression models (Gould et al. 2014), but as efficient computer programs are available to do the calculations this will rarely be an issue.

Some limitations of our analysis are now described. The subset of individuals with HAM-A scores recorded prior to the outcome had proportionately fewer outcome events than the full sample. This is likely because several offspring in our data set joined the study with a pre-existing diagnosis of major mood or bipolar disorder. These individuals had no prior measures of HAM-A scores, and so were excluded from the analysis.

This analysis contains only one repeatedly measured variable, but others such as depression scores may be important for prediction. Furthermore, clustering within families was ignored in the Joint model. A preferred approach would be to fit a Joint model with nested random effects in the longitudinal sub-model and a frailty term in the Cox sub-model. However available statistical software does not allow this. The effect of family clustering was investigated using a Cox model with frailty term (Table 3), which accounts for clustering and shown to be quite similar to the Cox analysis without frailty (Table 2). This suggests that there is a strong relationship between HAM-A scores and diagnosis of mood disorder, even after taking account of clustering. Note that this analysis using a Cox model does not properly account for measurement error.

Our sample size precluded the possibility of examining bipolar disorder alone as an outcome. In this cohort and in other high-risk offspring studies, it has been now well established that bipolar disorder debuts or onsets as major depression. Further, family studies have provided evidence that depressive disorders in family members of a proband with bipolar disorder, especially if recurrent MD with early onset (i.e. adolescence), are highly likely to represent the bipolar trait (Blacker et al. 1993). Therefore, depressive disorders in young people at confirmed risk for bipolar disorder is part of the bipolar phenotype. We have published on this several times (latest Duffy et al. 2014, 2018).

The *JM* package used to illustrate the Joint modelling framework in this paper is based on a maximum likelihood approach. Recent developments in Joint modelling employ Bayesian methods to avoid multivariate integration for less computational complexity (Gould et al. 2014). When multiple time-varying covariates are of interest, Bayesian methods may be preferred (Gould et al. 2014).

## Conclusions

In summary, anxiety both at the level of clinically significant symptoms and at the full-threshold syndrome level, is an important predictor of major mood disorder (major depression and bipolar disorder) in individuals at familial risk of developing bipolar disorder. Our analysis suggests that the magnitude of this association may be stronger than previously reported, due to the presence of measurement error in the time-varying covariate, which is not accounted for in the Cox model. We recommend the Joint modelling approach, as it takes account of measurement error and does not assume repeated measures remain constant between consecutive measurement times. These models can thus reduce bias and increase efficiency when modelling the effects of a repeatedly measured variable on the hazard of an event.

## Data Availability

Requests for access to de-identified data forming the basis of this analysis is available on request to the nominated principal investigator Dr. Anne Duffy.

## Abbreviations

DSM-IV: Diagnostic and Statistical Manual of Mental Disorders, 4th Edition
HAM-A: Hamilton Anxiety
KSADS-PL: Kiddie Schedule for Affective Disorders and Schizophrenia—Present and Lifetime Version
LVCF: Last Value Carried Forward
SADS-L: Schedule for Affective Disorders and Schizophrenia—Lifetime Version
SES: Socio-economical status
